# Hyaluronic acid spacer in prostate cancer radiotherapy: dosimetric effects, spacer stability and long-term toxicity and PRO in a phase II study

**DOI:** 10.1186/s13014-022-02197-x

**Published:** 2023-01-02

**Authors:** Ulrika Björeland, Kristina Notstam, Per Fransson, Karin Söderkvist, Lars Beckman, Joakim Jonsson, Tufve Nyholm, Anders Widmark, Camilla Thellenberg Karlsson

**Affiliations:** 1 Department of Radiation Sciences, Radiation Physics, Umeå University, 901 87 Umeå, Sweden; 2 Department of Radiation Sciences, Oncology, Umeå University, 901 87 Umeå, Sweden; 3 Department of Nursing, Umeå University, 901 87 Umeå, Sweden

**Keywords:** Prostate cancer, Radiotherapy, Rectal toxicity, Hyaluronic Acid

## Abstract

**Background:**

Perirectal spacers may be beneficial to reduce rectal side effects from radiotherapy (RT). Here, we present the impact of a hyaluronic acid (HA) perirectal spacer on rectal dose as well as spacer stability, long-term gastrointestinal (GI) and genitourinary (GU) toxicity and patient-reported outcome (PRO).

**Methods:**

In this phase II study 81 patients with low- and intermediate-risk prostate cancer received transrectal injections with HA before external beam RT (78 Gy in 39 fractions). The HA spacer was evaluated with MRI four times; before (MR0) and after HA-injection (MR1), at the middle (MR2) and at the end (MR3) of RT. GI and GU toxicity was assessed by physician for up to five years according to the RTOG scale. PROs were collected using the Swedish National Prostate Cancer Registry and Prostate cancer symptom scale questionnaires.

**Results:**

There was a significant reduction in rectal V70% (54.6 Gy) and V90% (70.2 Gy) between MR0 and MR1, as well as between MR0 to MR2 and MR3. From MR1 to MR2/MR3, HA thickness decreased with 28%/32% and CTV-rectum space with 19%/17% in the middle level. The cumulative late grade ≥ 2 GI toxicity at 5 years was 5% and the proportion of PRO moderate or severe overall bowel problems at 5 years follow-up was 12%. Cumulative late grade ≥ 2 GU toxicity at 5 years was 12% and moderate or severe overall urinary problems at 5 years were 10%.

**Conclusion:**

We show that the HA spacer reduced rectal dose and long-term toxicity.

**Supplementary Information:**

The online version contains supplementary material available at 10.1186/s13014-022-02197-x.

## Introduction

Rectal side effects are common after prostate cancer (PC) radiotherapy (RT). Higher radiation dose to the prostate can improve biochemical outcome, but dose escalation significantly increases the risk of treatment toxicity [1]. The anterior rectal wall is particularly vulnerable due to anatomical proximity to the prostate. Consequently, rectal complications are frequent, including loose stool, frequent defecation, urgency, rectal discharge or bleeding, and impaired quality of life (QoL) [2].

To reduce the rectal dose, a spacer can be inserted between the prostate and the anterior rectal wall to separate the rectum from the prostate. The first study using hyaluronic acid (HA) as a perirectal spacer by Prada et al. [3] has been followed by multiple studies with several different agents acting as spacers [4]. A majority of these studies are based on a polyethylene-glycol (PEG) hydrogel (SpaceOAR®) and suggest beneficial effects of spacers in reducing the rectal dose and, subsequently, late toxicity and QoL decline [5]. Other devices used for prostate-rectal separation are rectal balloon implants [6, 7] and collagen implants [8], both leading to decreased radiation doses to the rectum.

HA is a polysaccharide found in human tissues as a component of the connective tissue and previous studies have reported data on HA when used as a spacer in prostate RT [3, 9]. The quality, placement and thickness of the spacer are essential [5] and can influence rectum doses [10–12]. Despite many studies on perirectal spacers, most are small or lack long-term follow-up [5]. As a result, the impact on QoL and cost-effectiveness have been questioned, and the use of spacers in the clinic has not been universally accepted [13–15].

In this study, we present the impact of a HA perirectal spacer on rectal dose as well as spacer stability during RT with a five-year follow-up of patient-reported outcome (PRO) and gastrointestinal (GI)/genitourinary (GU) toxicity in conventionally fractionated definitive RT in prostate cancer.

## Methods

### Study design

In this Swedish single-center phase II study (NoHarm), 81 patients with low- and intermediate-risk PC were included between 2010 and 2016. Inclusion criteria were < 75 years of age, T1c-T2, Gleason ≤ 6 and PSA < 10 ng/mL, T1c- T3a with 1 or 2 of the following risk factors; T2c-T3a or Gleason ≥ 7 or PSA ≥ 10 ng/mL with WHO performance status 0–2 Additional file 1: Table S.1. No hormonal treatment was allowed. The patients received transrectal injections with HA as a spacer to separate the ventral rectal wall from the prostate. External beam RT, 2 Gy per fraction to 78 Gy, was given with 3DCRT or IMRT using gold seed fiducial markers as image guidance. A 7 mm isotropic margin around the prostate CTV was used to generate the PTV. Ethical approval was obtained from the ethical committee Dnr 09-227 M (2009-1433-31).

**Fig. 1:**
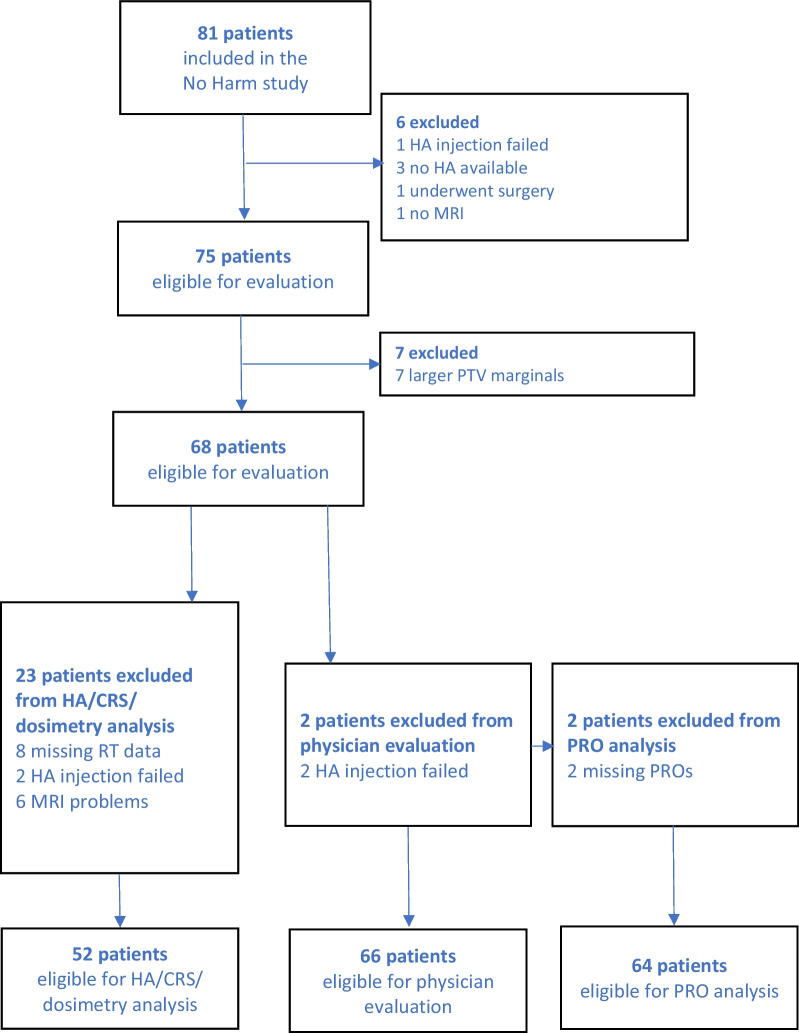
Inclusion/exclusion criteria. CRS = CTV-rectum space, HA = Hyaluronic acid, PRO = patient-reported outcome

Here, we present the results consisting of dosimetric/HA-spacer analysis and toxicity/PRO evaluation. Sixty-six patients were eligible for the toxicity analysis, 64 patients for PRO evaluation and 52 for dosimetric/HA-spacer evaluation; see Fig. 1.

### HA injection

The patients received 2–4 transrectal injections in the same session with approximately 15 ml of HA (Macrolane VRF 30, Q-Med/ Galderma, Uppsala, Sweden) between the ventral rectal wall and the Denonvilliers fascia on the prostate to achieve a space of > 10 mm between the two organs in prostate apex and base levels. The HA was injected into the perirectal space under transrectal ultrasound (TRUS) guidance with local anaesthetics, 10 mL lidocaine 1%.

### Toxicity and PRO analysis

Physician evaluated toxicity was assessed at baseline, end of RT, at 6, 12 and 18 months and 2, 3 and 5 years after the end of RT and graded according to the Radiation Therapy Oncology Group/European Organization for Research and Treatment of Cancer (RTOG/EORTC) scale [16]*.* PRO-questionnaires from the Swedish National Prostate Cancer Registry (NPCR) were used for monitoring bowel- and urinary problems and sexual function [17]. Two slightly different questionnaires (NPCR 2008, NPCR 2013) were used during the follow-up period due to an update of the questionnaires by the NPCR-group, and nine questions were evaluated. Example of a NPCR questionnaire is shown in Additional file 1 (SQ 1). Six additional questions from the prostate cancer symptom scale (PCSS) focused on bowel symptoms were also distributed each time [18, 19]. The PCSS is a validated PRO-questionnaire also used in other studies [20–22]. Questionnaires were distributed as follows; at the start of RT, mid-treatment and end of RT, and during follow-up at 3, 6, 9, 12, 18 months, and 2, 3, 4, and 5 years after the end of RT.

Due to the different scales of the questionnaires, the original scales of PCSS and NPCR 2008 were transformed from a 0–10-point numerical scale to a 4-point verbal scale, the same as were used in NPCR 2013. 0–1 were transformed to "No problem", 2–4 to "Small problems/few symptoms", 5–7 to "Moderate problems", and 8–10 to "Severe problems" [20]. To be able to calculate the mean score values, the 4-point scale was also converted to a scale of 0–100 according to the EORTC manual (a higher value refers to more symptoms or worse function) [23].

### Imaging, structures and registrations for dosimetry and HA-spacer

**Fig. 2:**
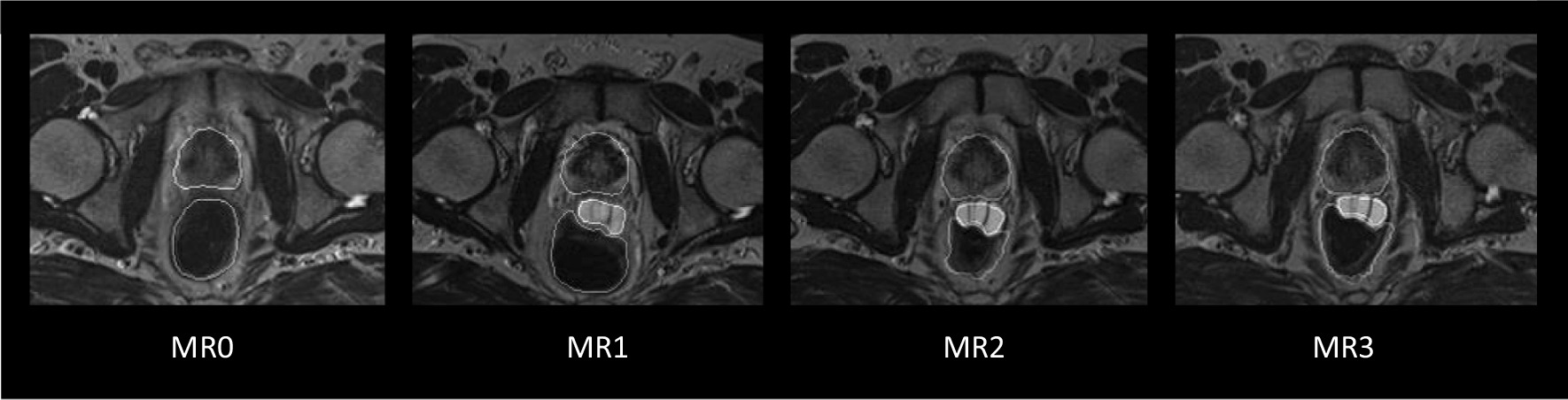
Example of T2w images in MR0/MR1/MR2/MR3 at the same level (base). HA is clearly visible between the rectum and CTV with a CRS 7/22/19/14 mm in MR0/MR1/MR2/MR3. HA thickness was 21/16/13 mm in MR1/MR2/MR3. MR1 was performed 1 day after HA, MR2 30 days and MR3 63 days after HA. CTV, rectum and HA are outlined

MRI scans (T2w-MRI) were achieved and evaluated before (MR0) and after (MR1/MR2/MR3) HA-injection (Fig. 2). The first scan with HA (MR1) was used in treatment planning (TP) for outlining the regions of interest (ROI). The second MRI (MR2) was scanned in the middle of the RT, and MR3 at the end of the RT. An Espree 1.5 T MRI, Siemens were used between 2010 and 2014, and after that, a Signa 3 T PET/MRI, General Electric. All scans were performed with a flat tabletop and similar positioning as during RT. CT and MR1 fiducial registration were performed as a basis for TP. The ROIs in MR1 were reviewed and modified in Oncentra by one radiation oncologist (KN) to ensure that all ROIs were outlined according to the same study protocol as for the HYPO-RT-PC trial [21, 24].

The HA, prostate CTV and rectum ROIs from TP were exported from Oncentra to MICE-Toolkit [25]. An automatic workflow using Elastix [26] implemented in MICE-Toolkit was created to outline HA, CTV and rectum ROIs in all other MRI scans. The outlined ROIs from MR1 were transferred to MR0, MR2 and MR3 using rigid and deformable image registration (R + DIR). Next, MR0, MR2 and MR3 images were co-registered with MR1. In this step, we used the same translations as in fiducial matching at treatment. This enabled the calculation of the rectum dose in MR0, MR2 and MR3 by using the TP from MR1. The following parameters were evaluated; rectal volumes receiving 90% (V90%), 80% (V80%), 70% (V70%), 60% (V60%), and 50% (V50%) of the prescribed dose. For HA evaluation in MR1/MR2/MR3, we measured the thickness and volume of the HA and CTV-rectum space (CRS) at three levels: middle (centre of CTV in cranial-caudal direction), base (10 mm cranial from the middle), and apex (10 mm caudal from the middle).

### Statistical analysis

The Wilcoxon signed-rank test was used to compare rectum doses based on MR1 to rectum doses based on MR0, MR2 and MR3. The differences in volume and shape/thickness of the ROIs were also tested with Wilcoxon. The tests were two-sided and considered significant if *p* < 0.05. Spearman's rank correlation coefficient test was performed to evaluate the thickness of HA compared to the CRS with a significance level of *p* < 0.05. Time to first late side-effect was calculated with the Kaplan-Meier method from the start of RT.

All statistical analyses were performed using the software SPSS statistics 27 (IBM, Armonk, New York, United States).

## Results

In total, 81 patients were included between 2010 and 2016. For the 68 patients eligible for evaluation in this study, the baseline characteristics reflect a mostly low-risk PC population with a median age of 68 years. 97% of RT was delivered with 3DCRT (Table 1). The tolerance of HA injection has been described in previous studies [3, 9]. In this study, 7 of 51 patients reported a feeling of anal fullness one week after HA. Two patients reported rectal bleeding, one patient reported fever and four patients frequent urination, not further specified (Additional file 1: Table S.2). The latter complications are similar to symptoms after transrectal fiducial marker implantation and considered to be acceptable complications [27, 28].

**Table 1:** Clinical characteristics and radiotherapy details

Characteristics	Value
*Age (years)*	
Median (range)	68 (52–79*)
*PSA (ng/ml)*	
Median (range)	7,7 (0.5–25)
*Gleason score (n)*	
6	56
7	10
8	2
*T stage (n)*	
T1c	47
T2	21
*RT technique (n)*	
3DCRT	66
VMAT/IMRT	2

^*^2 patients were included at age 76 and 79 but in those cases the study team

approved to continue with study treatment as planned

### HA spacer and CTV-rectum space

**Fig. 3:**
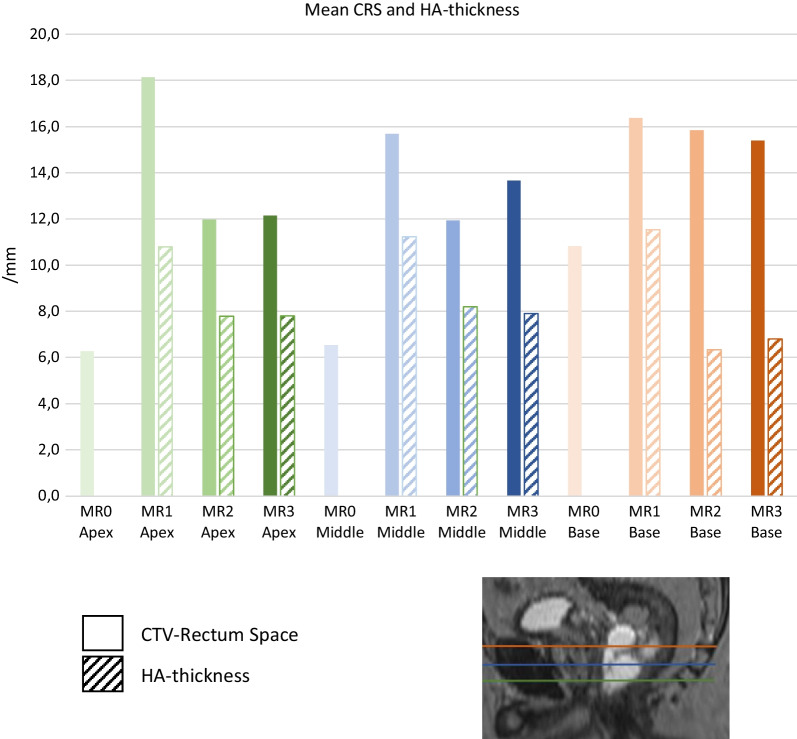
Mean CTV-Rectum Space (CRS) and HA thickness in MR0, MR1,MR2 and MR3

The mean injected HA was 16 ± 4 [range 8–24] cm^3^ to achieve 10 mm CRS in prostate base and apex levels for the 52 patients in this part of the evaluation, see Fig. 1. Measured HA volumes in MRI were 15% lower than injected volumes. The volume estimation from MRI images depends on both outlining of HA and image resolution, and this could explain the difference. In addition, some of the injected HA was unfortunately deposited in rectum for two patients. The median time between HA injection and MR1 was one day [range 0–5]. The HA thickness decreased significantly from MR1 to MR2/MR3, but no significant difference was seen between MR2 and MR3, see Fig. 3.

HA caused a significant increase in CRS from MR0 to MR1/MR2/MR3. A CRS > 10 mm in the prostate base, middle and apex levels was achieved in 35 patients (67%) in MR1, 23 patients (44%) in MR2 and 26 patients (50%) in MR3. Even without HA, six patients (12%) had a CRS > 10 mm in all three levels due to anatomical variations. From MR0 to MR1, the CRS increased by 114%. CRS decreased by 19% in MR2 and 17% in MR3 compared to MR1 in the middle level. Further, HA thickness decreased by 28% in MR2 and 32% in MR3 compared to MR1 in the middle level. See Additional file 1: Table S.2 for all other levels. HA thickness in the middle and apex levels showed a significant moderate to strong correlation to CRS in MR1. See study images in Fig. 2 for visualisation of HA and CRS. However, CRS were significantly smaller in MR2/MR3 compared to MR1, but no significant difference in CRS was seen between MR2 and MR3.

For the CRS variation with V90% combined with grade 2 and 3 rectal toxicity see Additional file 1: Fig. S.1 A–C. For summary of dosimetry and rectal, CTV, HA and CRS characteristics, see Additional file 1: Table S.3. For all significant correlations, see Additional file 1: Table S.4.

### Dosimetry

The rectal dose constraints for V90% < 15% were fulfilled in 50 out of 52 (96%) patients in MR1 and 47 (90%) patients in MR2 and MR3. Without HA, 46 (88%) patients matched the V90% criteria.

**Fig. 4:**
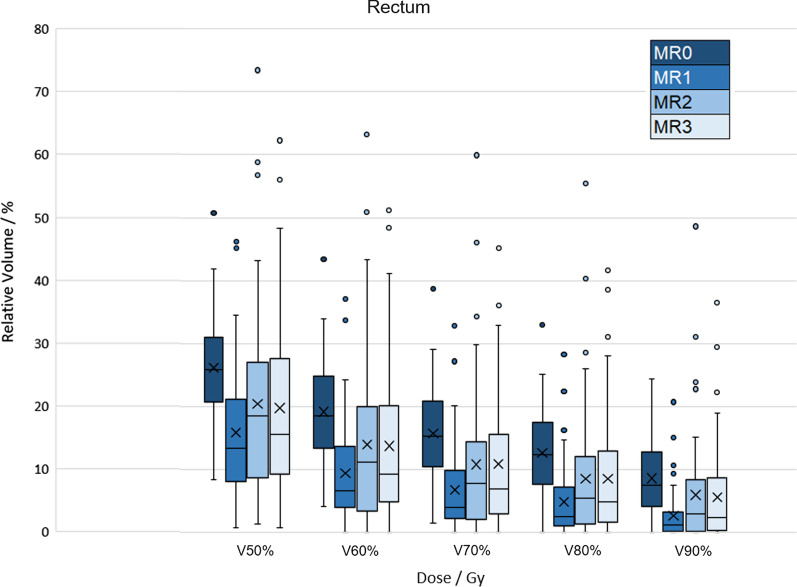
Relative rectal volumes receiving 90% (V90%), 80% (V80%), 70% (V70%), 60% (V60%), and 50% (V50%) of the prescribed dose (78 Gy)

Rectal V50%-V90% were significantly reduced in MR1–MR3 compared to MR0. The reduction in V70%/V90% was 56%/69% from MR0 to MR1. Furthermore, the reduction was 31%/30% from MR0 to MR2 and 31%/34% from MR0 to MR3, respectively (Additional file 1: Table S.2). Though the reduction was not as pronounced in MR2 and MR3 than in MR1, it was still significant for all three occasions compared to MR0. No difference was seen for V50%-V90% between MR2 to MR3 (Fig. 4). A weak to moderate significant correlation was found between V90% and CRS. See Additional file 1: Fig S.1. A–C for variations during RT in combination with CRS and toxicity grades.

### Physician-recorded toxicity graded by RTOG/EORTC

**Fig. 5:**
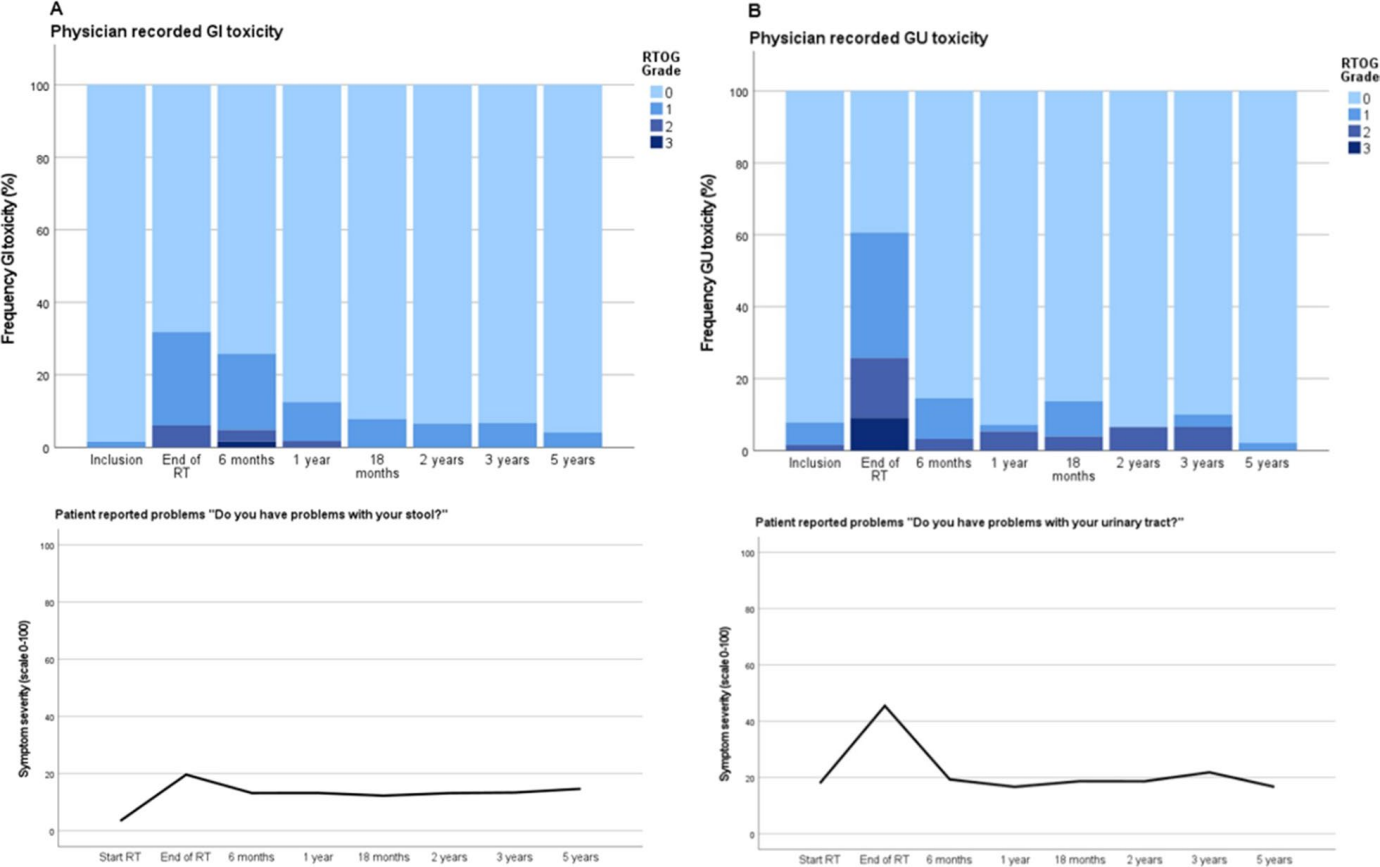
**A** GI toxicity (RTOG) and patient reported outcome (measured with the question "Do you have problems with your stool?"). **B** GU toxicity (RTOG) and patient reported outcome (measured with the question "Do you have problems with your urinary tract?")

**Fig. 6:**
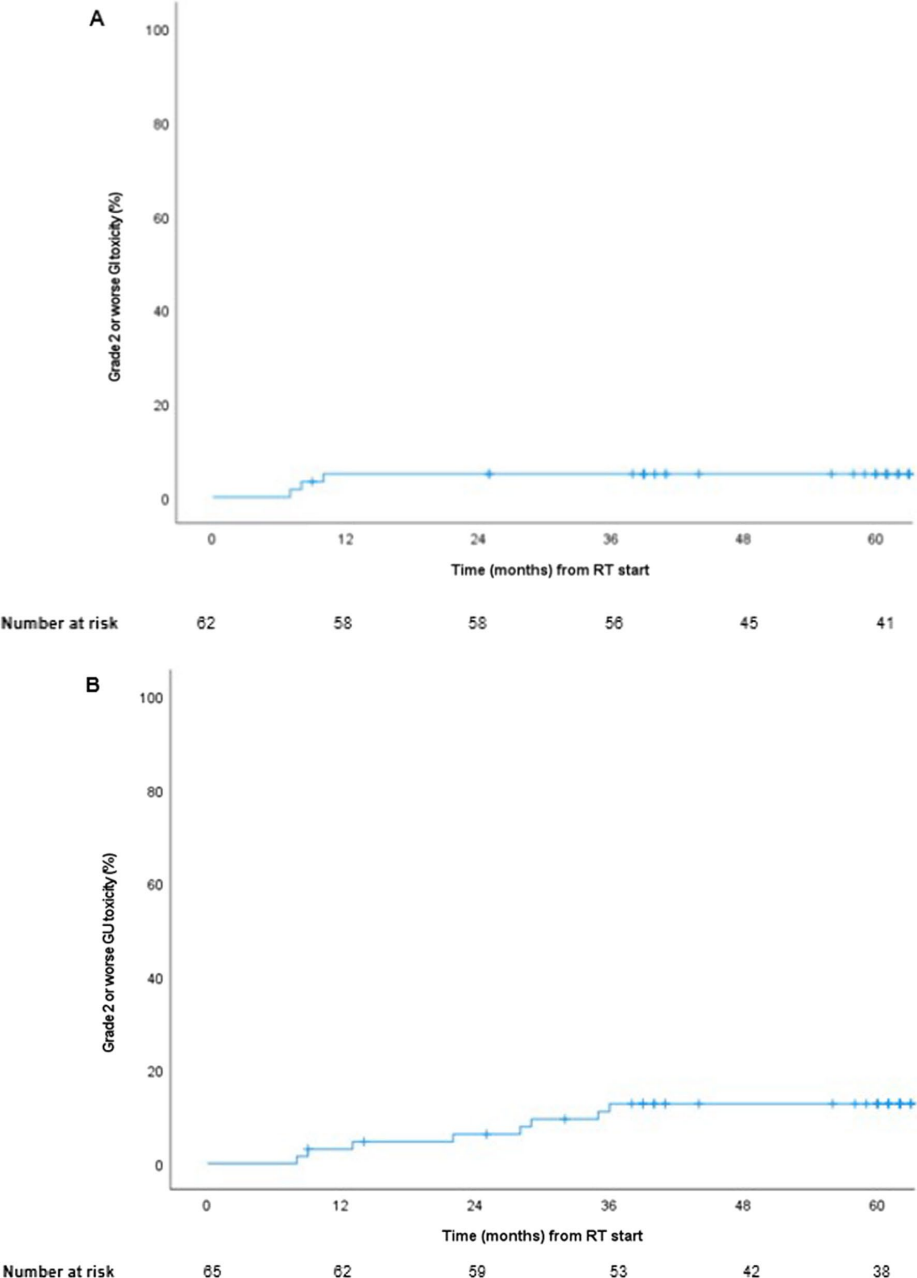
Cumulative incidence of physician-reported late GI (A) and GU (B) toxicity grade 2 or worse according to the RTOG scale

Toxicity was graded according to the RTOG/EORTC scale [16]. At the end of RT, 17 patients (26%) experienced acute grade 1 GI toxicity (i.e., increased frequency or change in quality of bowel habits or rectal discomfort not requiring medication). Four patients (6%) experienced grade 2 GI toxicity (i.e., diarrhea requiring parasympatholytic drugs or mucous discharge or rectal/abdominal pain requiring analgesics). No acute grade ≥ 3 GI toxicity was reported. At six months, the proportion of grade 1 GI toxicity was 21%, grade 2 3%, and one patient (2%) experienced grade 3 toxicity (proctitis). After 18 months, no grade ≥ 2 GI toxicity was observed (Fig. 5A). The estimated cumulative late grade ≥ 2 GI toxicity rate at both 2 and 5-year follow-up were 5% (Fig. 6A).

The frequency of grade 2 GU toxicity at the end of RT was 17%, and grade 3 was 9%. At 2 and 5 years, late grade ≥ 2 toxicity was 7% and 0%. Only 2% experienced grade ≥ 1 toxicity at 5 years follow-up (Fig. 5B). The cumulative late grade ≥ 2 GU toxicity was 6% at 2-year follow-up and 12% at 5-year follow-up (Fig. 6B).

### PROs

In total, 64 patients were included for PRO evaluation (Fig. 1). The proportion of missing questionnaires at the evaluated follow-up times varied. At 5 years, 64% (41/64) of the NPCR questionnaires and 36/64 (56%) of the PCSS questionnaires could be evaluated.

The prevalence of patients reporting moderate or severe overall bowel problems (≥ 67 on the 0–100 scale) measured with the question "Do you have problems with your stool?" at RT start, end of RT and 5 years was 0%, 18% and 12%. Mean scores (scale 0–100; higher value refers to more symptoms or worse function) for overall bowel bother was 3.4 at the start of RT, 13.1 at 2 years and 14.6 at 5 years (Fig. 5A). At RT start and at 5 years follow-up, the prevalence of patients reporting moderate/severe problems with stool leakage was 0% and 2%, mucus in stool 0% and 5%, blood in stool 0% and 0%, flatulence 15% and 11%, bowel cramps 0% and 6%, limitation in daily activity 0 and 2%.

The proportion of patients reporting moderate or severe overall urinary problems ("Do you have problems with your urinary tract?") at RT start, end of RT and 5 years was 15%, 42% and 10%. Mean scores (scale 0–100) for overall urinary bother were 18.0 at start of RT, 18.6 at 2 years and 16.7 at 5 years (Fig. 5B). At start and at 5 years follow-up, patients reported moderate/severe problems with weak stream were 13% and 24%, urgency 3% and 20%, limitation in daily activity 3% and 7%, respectively.

Detailed results from the questionnaires are shown in Additional file 1: Tables S.5–S.8.

## Discussion

Our study confirmed reduced rectum doses shown by several studies using mainly PEG-spacers [3, 12, 29–31]. Today, the most common spacer used is PEG hydrogel (SpaceOAR) which is well documented [32]. It is injected as a liquid and polymerised in situ into a soft gel in less than 10 s [4]. SpaceOAR was found stable during RT [33], and a time frame of one day between injection and TP was acceptable according to volume and dice similarity coefficient [34]. The spacer used in this study, HA, is injected as a gel. Because of its viscosity, HA may not distribute evenly [4] and several injections are often used [9]. Further, in laboratory findings, the HA viscosity decreases during irradiation [35].

In this study, the CRS decreased during RT, resulting in a larger relative rectum volume receiving dose in MR2 and MR3 than in MR1. This might be due to changes in HA viscosity, size or in the position of the spacer. The MRI used for treatment planning (MR1) was, in median, performed the day after the application of HA with a range from 0 to 5 days. Our results indicate that the spacer is not stable at this time, but it is between MR2 and MR3. Since no MRI was performed between MR1 and MR2, we can not determine when stabilisation of the spacer occurs. However, HA still significantly reduced the rectal V50%-V90%.

Susil et al. [36] show that a mean CRS of 10 mm was sufficient for V90% dose reduction, and a larger distance did not result in lower rectal doses. In our study, the mean CRS was over 13 mm, and the rectal V90% decreased from 9% (MR0) to 6% (MR2 and MR3). These results are comparable to Susil et al. [36]. Further, Pinkawa et al. [37] reported a rectal V90% of 13% without a spacer and 4% with a spacer, similar to our findings.

We noticed that the patient with rectal grade 3 toxicity fulfilled the rectum criteria for V90% of 15% in MR1 but not in MR2 and MR3. Surprisingly, the CRS were around 10 mm in all levels in MR1/MR2/MR3, so the HA was separating the rectum from the prostate as intended. Relating CRS to rectal dose or side effects is complex, the dose optimisation is based on one image occasion, and our data shows a variation in rectal dose during RT, see Fig. 4. This variation during RT can be of importance, especially in hypofractionated and dose escalated PC RT.

A strength of the imaging part of the study is the comprehensive MRI protocol enabling evaluation of the HA spacer over time. Possible limitations could be that we measured CRS in only three levels and assumed that the CTV centre did not vary in base-apex extension during RT. Moreover, we used automatic registrations to transfer ROIs from MR1 to MR2/MR3, resulting in more edgy structures compared to ROIs delineated by a radiation oncologist. This is because manual outlining in the clinic tends to smooth edges; registrations operate only on pixel intensities. In addition, the form and placement changes of the HA-spacer can lead to the underestimation of HA-thickness.

Uhl et al. [38] reported low GI acute toxicity and only 4.3% late grade 1 with no late grade ≥ 2 GI toxicity at 6 and 12 months follow-up with a spacer. Those patients received conventionally fractionated RT to a dose of 78 Gy delivered with IMRT technique. Whalley et al. [12] described late rectal toxicity similar to our results in a hydrogel spacer study to 80 Gy in 40 fractions with IMRT or VMAT. Our study has a long follow-up time but no control arm to compare toxicity. Studies have shown that the risk of toxicity is reduced with IMRT compared to 3DCRT [39, 40] and most of the studies referred to here are based on the use of IMRT. In this study, 3DCRT was the most common RT technique, and for this reason, it can be challenging to compare the toxicity with those results. However, a comparison with the control arm in the ultra-hypofractionation study HYPO-RT-PC is relevant, as the total dose and dose per fraction were the same, a similar radiotherapy technique was used, and no hormonal treatment was allowed. Furthermore, the HYPO-RT-PC study also used PRO questions from the PCSS questionnaire and had a similar time-period of inclusion as our site. On the other hand, there is a higher proportion of intermediate-high-risk tumours in the HYPO-RT-PC [20, 21].

The proportion with GI toxicity grade ≥ 1 at 2- and 5-years follow-up seem to be lower in this study than in the HYPO-RT-PC control arm (7% vs. 18% and 4% vs. 15%). Furthermore, the grade ≥ 2 GI toxicity at 2 and 5 years were slightly lower here than in the HYPO control arm, as well as the cumulative late grade ≥ 2 GI toxicity [21]. This is in line with previous studies that have shown a reduction in late grade ≥ 1 GI toxicity with a spacer [12, 41]. Compared to the HYPO control arm, the proportion of grade ≥ 1 GU toxicity at 5 years was lower (2% vs 22%) and, similarly, the late grade ≥ 2 toxicity (0 vs. 5%) [21]. GU toxicity decline with a spacer in another study has been more modest except regarding incontinence [41]. Taken together, the HA spacer could play a role in the mentioned differences, but other factors could influence the results since it is not a matched control group.

Even though we noted a reduced rectal dose, there were no big differences in the PROs compared to results from the HYPO-RT-PC [21]. The proportion of patients that reported moderate/severe overall GI-bothers in the PROs at RT start/5 years was about the same as in the HYPO study at baseline/6 years. Similarly, no large differences in specific GI symptoms were seen, e.g., moderate/severe problems with stool leakage, mucus and blood in stool at 5 years. Furthermore, the proportion of patients who reported moderate or severe overall urinary problems was quite similar to the HYPO-RT-PC control arm [21].

There are limitations in this study regarding the PROs. For example, different scales were used in the follow-up questionnaires since the questionnaires changed during the long inclusion period. Furthermore, there was a considerable loss of questionnaires in the study. This may explain the possible difference in outcomes between physician-evaluated side effects and patient-reported ones. On the other hand, there are weaknesses with the RTOG scale that could lead to underestimating late bowel toxicity [42]*.* Furthermore, data indicate that physician-reported toxicity can underestimate the frequency and severity of toxicity compared with results from PROs [43]. Other limitations are the conventionally fractionated schedule with 3DCRT technique which is not yet longer in use and the non-randomised setting which make it difficult to compare this series with others.

In this study a mostly low-risk PC population treated with conventionally fractionated RT was included. This group may initially have a relatively low risk of toxicity and limited benefit from a spacer. However, rectal spacing could probably be particularly beneficial in ultra-hypofractionated RT as well as in high-risk PC treated with dose-escalated RT since doses to the rectum potentially could be higher. Similarly, Hutchinson et al.[13] described spacer cost-effectiveness when used in high-dose SBRT but not in conformal RT [13]. Furthermore, decision rules for selecting which patients will benefit most from spacer implantation are being developed based on clinical risk factors [44].

## Conclusions

This study shows that injection of a perirectal HA spacer resulted in a reduced rectal dose and low Gl and GU 5-year toxicity. These results are similar to findings from other spacer studies. Furthermore, we show spacer stability after approximately 30 days and throughout the treatment but questions about how early the HA reaches stability remain.

## Supplementary Information

**Additional file 1**: Supplementary materials.

## Data Availability

The datasets used and/or analysed during the current study are available from the corresponding author on reasonable request. Anonymized DICOM images and structures may be made available after required patient consent to international data sharing.

## Abbreviations

CRS: CTV-rectum space
CTV: Clinical target volume
EORTC: European Organization for Research and Treatment of Cancer
GI: Gastrointestinal
GU: Genitourinary
HA: Hyaluronic acid
MR0: Baseline MRI without HA
MR1: First MRI with HA
MR2: Second MRI with HA
MR3: Third MRI with HA
MRI: Magnetic resonance imaging
NPCR: Swedish National Prostate Cancer Registry
PC: Prostate cancer
PCSS: Prostate cancer symptom scale
PEG: Polyethylene-glycol
PRO: Patient-reported outcome
ROI: Regions of interest
RT: Radiotherapy
RTOG: Radiation Therapy Oncology Group
TP: Treatment planning
TRUS: Transrectal ultrasound
