# Supplementary material for: Hyaluronic acid spacer in prostate cancer radiotherapy: dosimetric effects, spacer stability and long-term toxicity and PRO in a phase II study
Source: Radiat Oncol. 2023 Jan 2;18:1. doi: 10.1186/s13014-022-02197-x (PMC9809044; doi:10.1186/s13014-022-02197-x)
Supplement: Supplementary file 1 — Additional file 1: Supplementary materials. [file 13014_2022_2197_MOESM1_ESM.pdf]

Supplementary materials

Table S.1 Inclusion/exclusion criteria in the No Harm study.

Inclusion criteria:
1. Men < 75 years of age and, as judged by the doctor, a life expectancy of 10 years (except for cancer) at time of inclusion with performance status WHO 0-2.
2. Patients with a histologically verified prostatic cancer.
3. Patients with low risk prostate cancer (T1c-T2, and Gleason ≤ 6 and PSA < 10). Patients with intermediate risk prostatic cancer of clinical category T1c- T3a with 1 or 2 of the following risk factors; T2c-T3a or Gleason ≥ 7 or PSA ≥ 10; according to the TNM classification system UICC 2002.
4. Patients should be lymph node negative according to the definition above, i.e. staging
5. Patients must have signed informed consent

Exclusion criteria:
1. Patients who earlier have undergone any other treatment for prostatic cancer, including Neo-adjuvant hormonal treatment.
2. Patients unable to co-operate or suffering from any other form of disease that would interfere with the planned treatment (e.g. colitis)
3. Patients with previous diagnosis of other malignant disease. Exceptions could be made for basal cell carcinoma of the skin or progression free survival at least 10 years after any previous tumour.
4. Any condition that prevent implantation of spacer (i.e. lower abdominal surgery, patients with haemofili).

Table S.2. Reported events one week after HA injection.

**Reported events one week after HA**	**Number of patients (n=51)**
Nothing unusual	36(70.6%)
Feeling of anal fullness	7(13.7%)
Pain with HA	1(2.0%)
Fever after HA	1(2.0%)
Rectal bleeding > 3 days	2(3.9%)
Urinary Frequency	4(7.8%)

Table S.3. Summary of dosimetry and rectal, CTV, HA and CRS characteristics.

	MR0	MR1	MR2	MR3	Wilcoxon p-values and change
Rectum characteristics					
Mean volume / cm3 mean±sd [range]	74±40 [29-253]	68±28 [31-141]	76±30 [336-175]	78±35 [41-215]	MR0-MR1:0.495 (-8%)<br>MR1-MR2: 0.003 (13%)<br>MR1-MR3: 0.002 (16%)<br>MR2-MR3: 0.807 (3%)
Relative volume V70% (54.6 Gy) /% mean±sd [range]	16±8 [2-39]	7±7 [0-33]	11±12 [0-60]	11±11 [0-45]	MR0-MR1: <0.0001 (-56%)<br>MR0-MR2: <0.0001 (-31%)<br>MR0-MR3: 0.001 (-31%)<br>MR1-MR2: 0.002 (59%)<br>MR1-MR3: 0.001 (59%)<br>MR2-MR3: 0.855 (0.5%)
Relative volume V90% (70.2 Gy) /% mean±sd [range]	9±6 [0-24]	3±4 [0-21]	6±9 [0-49]	6±8 [0-36]	MR0-MR1: <0.0001 (-69%)<br>MR0-MR2: 0.001 (-30%)<br>MR0-MR3: 0.002 (-34%)<br>MR1-MR2: 0.001 (24%)<br>MR1-MR3: <0.0001 (12%)<br>MR2-MR3: 0.483 (-5%)
Correlation: Rectum volume vs. (V70% or V90 %)	Not Sig	Not Sig	Not Sig	Not Sig	
Correlation: HA thickness vs. (V70% or V90 %)		Not Sig	Not Sig	Not Sig	
CTV					
Mean volume / cm3	56±24 [24-124]	58±19 [28-126]	59±19 [31-115]	55±20 [24-135]	MR0-MR1:0.002 (3%)<br>MR1-MR2: 0.003 (3%)<br>MR1-MR3: 0.003 (-4%)<br>MR2-MR3: <0.0001 (-6%)
HA characteristics					
Injected or measured volume / cm3 mean±sd [range]	Injected volume: 16±4 [8-24]	13±5 [6-30]	10±5 [1-26]	9±5 [1-24]	Inj.vol- MR1: 0.001 (-15%)<br>MR1-MR2: <0.0001 (-27%)<br>MR1-MR3: <0.0001 (-30%)<br>MR2-MR3: 0.006 (-4%)
Median time in days from HA [range]	Median time in days between HA and RT start: 7 [2-33]	1 [0-5]	34 [27-62]	62 [55-85]	
HA thickness Base / mm mean±sd [range]		13±5 [4-27]	9 ±5 [1-17]	8±5 [1-16]	MR1-MR2: < 0.001 (-31%)<br>MR1-MR3: < 0.001 (-35%)<br>MR2-MR3: 0.849 (-6%)
HA thickness Middle / mm mean±sd [range]		12±5 [5-22]	9 ±4 [1-24]	8±4 [1-26]	MR1-MR2: < 0.001 (-28%)<br>MR1-MR3: < 0.001 (-32%)<br>MR2-MR3: 0.444 (-6%)
HA thickness Apex / mm mean±sd [range]		13±6 [2-31]	8±4 [1-17]	8±4 [1-17]	MR1-MR2: < 0.001 (-41%)<br>MR1-MR3: < 0.001 (-41%)<br>MR2-MR3: 0.660 (0%)

	MR0	MR1	MR2	MR3	Wilcoxon p-values and change
CTV-Rectum space (CRS)					
Base /mm<br><br>n= number of patients	11±8, n=52<br><br>[n=24, CRS>10 mm]	19±8, n=52<br><br>[n=48, CRS>10 mm]	16±6, n=52<br><br>[n=42, CRS>10 mm]	16±6, n=52<br><br>[n=39, CRS>10 mm]	MR0-MR1: < 0.001 (69%)<br>MR0-MR2: < 0.001 (45%)<br>MR0-MR3: < 0.001 (40%)<br>MR1-MR2: 0.001 (-14%)<br>MR1-MR3: < 0.001 (-17%)<br>MR2-MR3: 0.563 (-4%)
Middle /mm<br><br>n= number of patients	7±5, n=52<br><br>[n= 11, CRS>10 mm]	16±5, n=52<br><br>[n=45, CRS>10 mm]	13±5, n=52<br><br>[n=36, CRS>10 mm]	13±5, n=52<br><br>[n=38, CRS>10 mm]	MR0-MR1: < 0.001 (114%)<br>MR0-MR2: < 0.001 (74%)<br>MR0-MR3: < 0.001 (80%)<br>MR1-MR2: < 0.001 (-19%)<br>MR1-MR3: < 0.001 (-17%)<br>MR2-MR3: 0.716 (3%)
Apex /mm<br><br>n= number of patients	7±4, n=52<br><br>[n=8 CRS>10 mm]	16±6, n=52<br><br>[n=39 CRS>10 mm]	13±5, n=52<br><br>[n=33 CRS>10 mm]	13±6, n=52<br><br>[n=34 CRS>10 mm]	MR0-MR1: < 0.001 (149%)<br>MR0-MR2: < 0.001 (100%)<br>MR0-MR3: < 0.001 (106%)<br>MR1-MR2: < 0.001 (-19%)<br>MR1-MR3: < 0.001 (-17%)<br>MR2-MR3: 0.489 (3%)

Table S.4. Significant Correlations p<0.01. HA= Hyaluronic acid, CRS= CTV-rectum space

	MR0			MR1			MR2			MR3		
	CRS Base	CRS Middle	CRS Apex	CRS Base	CRS Middle	CRS Apex	CRS Base	CRS Middle	CRS Apex	CRS Base	CRS Middle	CRS Apex
HA thickness Apex						0.64			0.60			0.78
HA thickness Middle					0.71			0.49				
HA thickness Base				0.48								
Rel. rectum V70 % (54,6 Gy)			-0.39	-0.40	-0.53	-0.43			-0.41	-0.40		-0.40
Rel. rectum V90 % (70,2 Gy)			-0.46		-0.49				-0.40	-0.39		-0.38

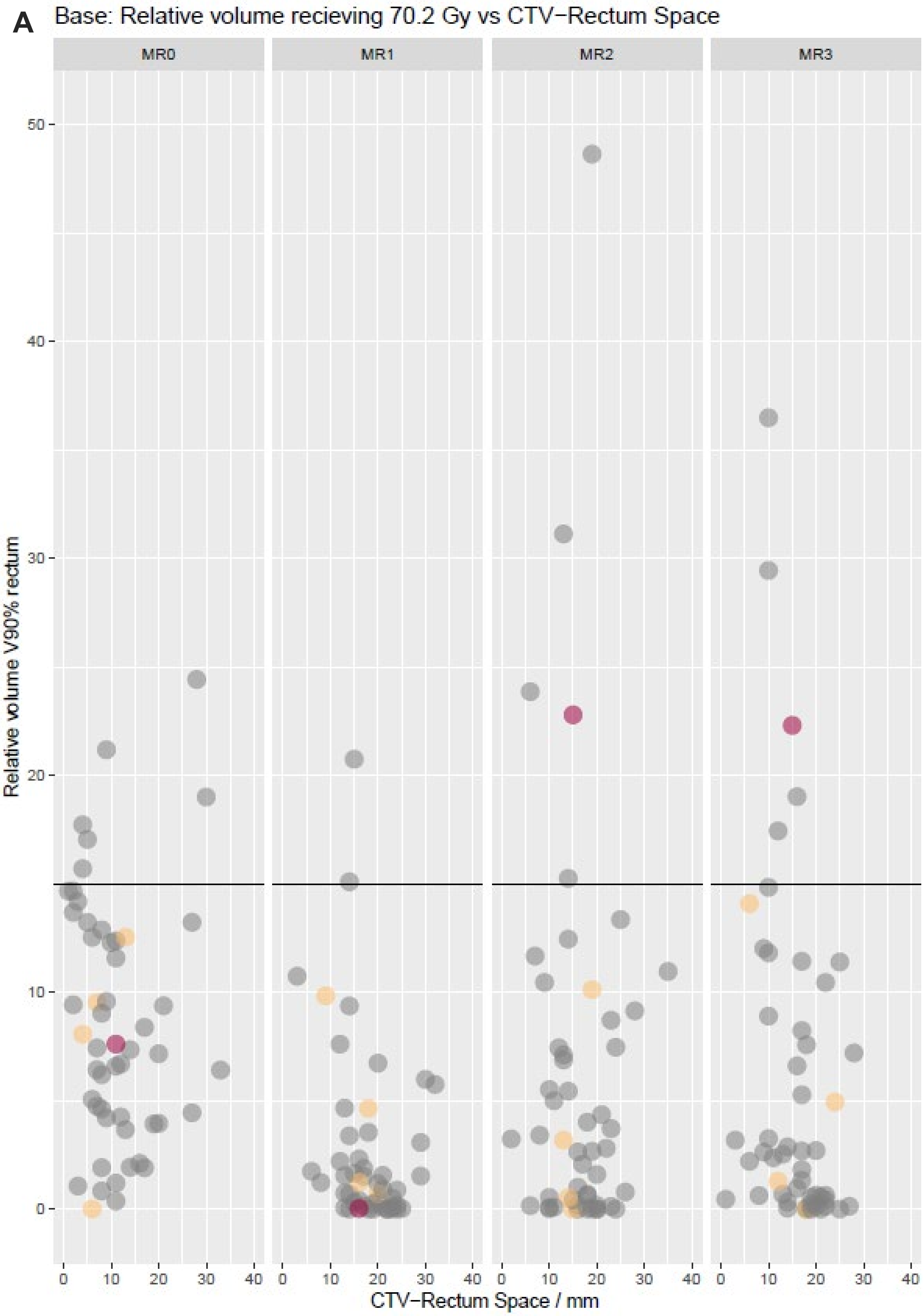

Fig. S.1. A. Base level. CTV to Rectum Space in three levels with respect to the relative rectum volume receiving V90% (70.2 Gy) dose. Yellow dot= Grade 2 rectal toxicity, red dot = Grade 3 rectal toxicity. Horizontal line indicates the 15% rectum criteria.

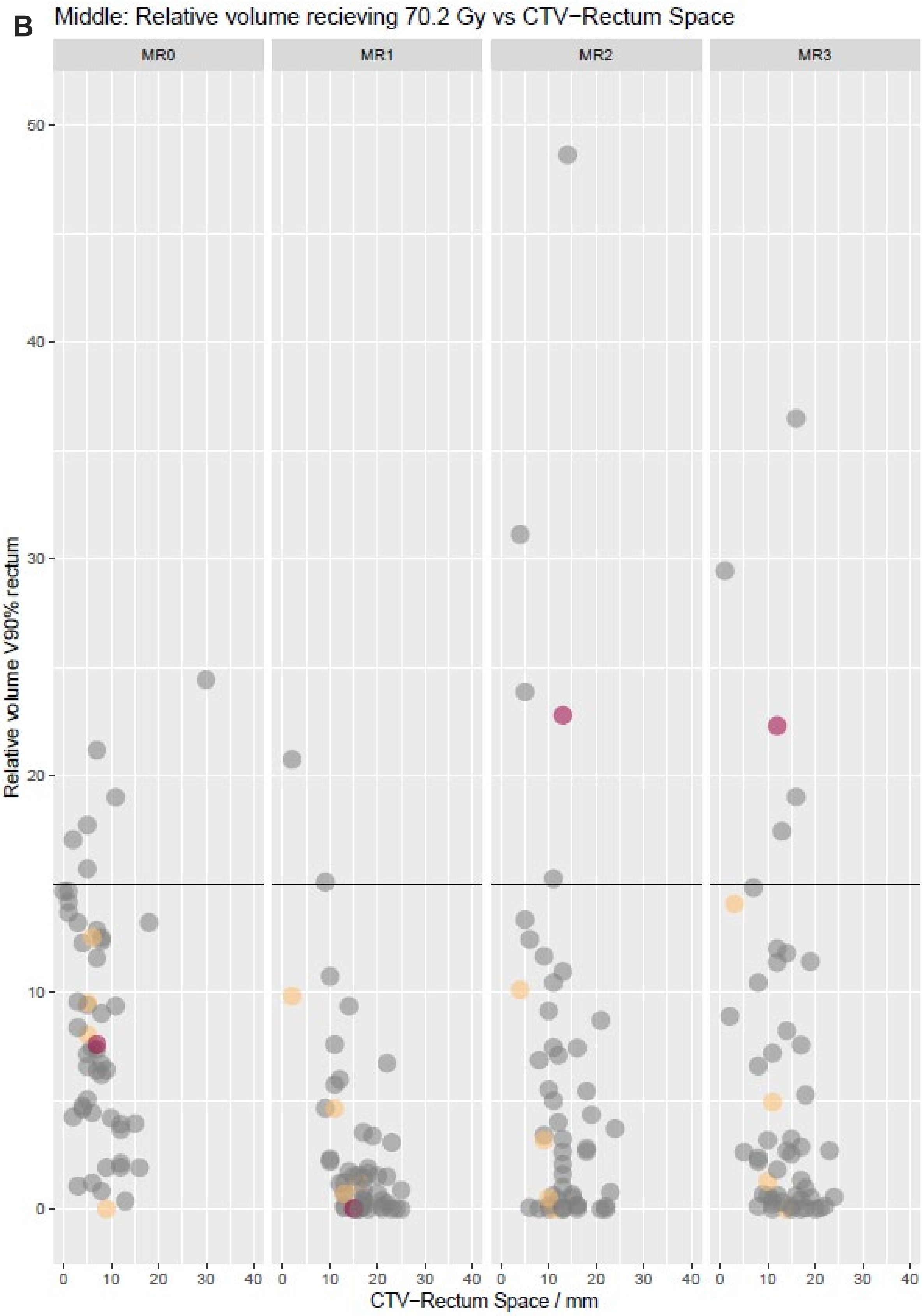

Fig. S.1. B. Middle level. CTV to Rectum Space in three levels with respect to the relative rectum volume receiving V90% (70.2 Gy) dose. Yellow dot= Grade 2 rectal toxicity, red dot = Grade 3 rectal toxicity. Horizontal line indicates the 15% rectum criteria.

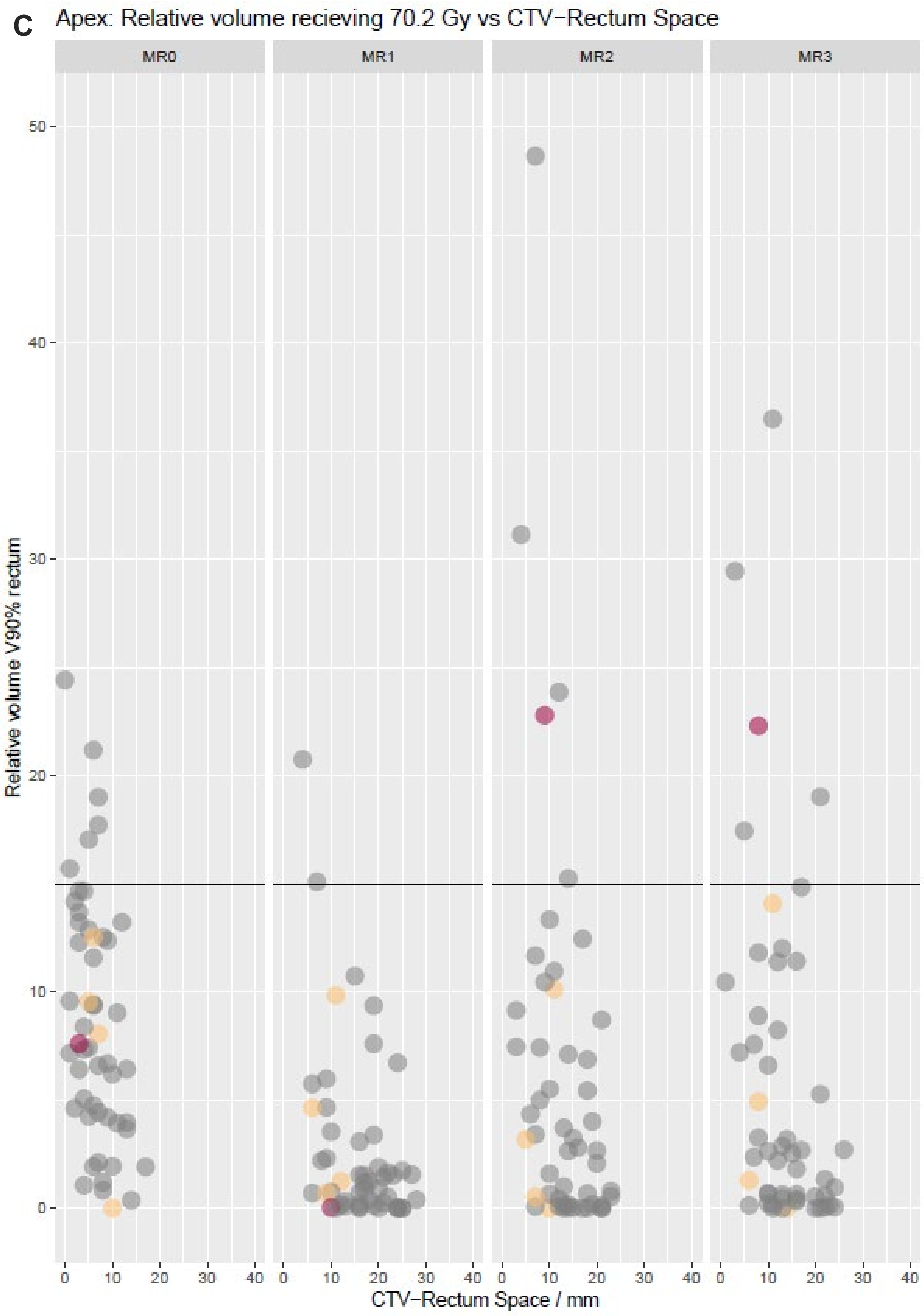

Fig. S.1. C. Apex level. CTV to Rectum Space in three levels with respect to the relative rectum volume receiving V90% (70.2 Gy) dose. Yellow dot= Grade 2 rectal toxicity, red dot = Grade 3 rectal toxicity. Horizontal line indicates the 15% rectum criteria.

Table S.5. The prevalence of patients reporting no/little and moderate/severe overall bowel problems (≥67 on the 0-100 scale) measured with the question "Do you have problems with your stool?"

	No/Little problems	Moderate/Severe problems	Total
Start RT	39 (100%)	0 (0%)	39 (100%)
End of RT	28 (82%)	6 (18%)	34 (100%)
6 months	35 (92%)	3 (8%)	38 (100%)
1 year	44 (92%)	4 (8%)	48 (100%)
18 months	45 (92%)	4 (8%)	49 (100%)
2 years	31 (94%)	2 (6%)	33 (100%)
3 years	50 (91%)	5 (9%)	55 (100%)
5 years	36 (88%)	5 (12%)	41 (100%)

Table S.6. The prevalence of patients reporting moderate/severe bowel problems at RT start and at 5 years follow-up.

	RT start	5 years
Stool leakage	0/39 (0%)	1/41 (2%)
Mucus	0/39 (0%)	2/41 (5%)
Blood in stool	0/40 (0%)	0/41 (0%)
Flatulence	5/33 (15%)	4/36 (11%)
Bowel cramps	0/25 (0%)	2/36 (6%)
Limitation in daily activity	0/39 (0%)	1/41 (2%)

Table S.7. The prevalence of patients reporting no/little and moderate/severe overall urinary problems (≥67 on the 0-100 scale) measured with the question "Do you have problems with your urinary tract?"

	No/Little problems	Moderate/Severe problems	Total
Start RT	33 (85%)	6 (15%)	39 (100%)
End of RT	19 (58%)	14 (42%)	33 (100%)
6 months	33 (87%)	5 (13%)	38 (100%)
1 year	43 (90%)	5 (19%)	48 (100%)
18 months	43 (86%)	7 (14%)	50 (100%)
2 years	29 (85%)	5 (15%)	34 (100%)
3 years	45 (82%)	10 (18%)	55 (100%)
5 years	36 (90%)	4 (10%)	40 (100%)

Table S.8. The prevalence of patients reporting moderate/severe urinary problems at RT start and at 5 years follow-up.

	RT start	5 years
Weak stream	5/39 (13%)	10/41 (24%)
Urgency	1/39 (3%)	8/41 (20%)
Limitation in daily activity	1/39 (3%)	3/41 (7%)

S.Q.1 NPCR questionnaire

NPCR Nationella prostatacancerregistret — REGIONALA CANCERCENTRUM I SAMVERKAN

To be filled in by the registry secretariat

Patient no: ☐☐☐☐☐☐ **Time:** ☐☐☐

Date when questionnaire was completed: (Year- Month- Day):

☐☐☐☐-☐☐-☐☐

e.g. 2016-05-15

Person Identity Number (Year-Month-Day- Control number)

☐☐☐☐-☐☐-☐☐-☐☐☐☐

e.g. 1945-06-28-8519

Questions about information/participation

	Not at all	Some	Moderately	Much/Very
1. Do you feel that you participated in the decisions about your care and treatment as much as you wanted?	☐	☐	☐	☐
2. Do you have a named contact nurse?	☐Yes	☐No	☐ I do not know	

During your present illness or treatment, how much information have you received about:

	Not at all satisfying	To some extent satisfying	Moderately satisfying	Very satisfying
3. Possible side effects of your treatment?	☐	☐	☐	☐
4. The effect of the treatment on your sex life?	☐	☐	☐	☐

General questions about your health:

5. How would you describe your health?	Very poor	☐ 1	☐ 2	☐ 3	☐ 4	☐ 5	☐ 6	☐ 7	Excellent
6. How would you describe your quality of life?	Very poor	☐ 1	☐ 2	☐ 3	☐ 4	☐ 5	☐ 6	☐ 7	Excellent

	Not at all	A little	Moderately	Much/Very
7. How much does your prostate cancer illness or treatment affect your daily activities?	☐	☐	☐	☐
8. Do you feel worried?	☐	☐	☐	☐

Questions about your urination, ***during the past month:***

	Not at all	A little	Moderately	Much/Very
9. Are you happy with how your urination function?	☐	☐	☐	☐
10. Is your urine stream weak?	☐	☐	☐	☐
11. Do you experience urinary urgency?	☐	☐	☐	☐
12. How much urine leakage do you experience?	☐	☐	☐	☐

13. Do you have urine leakage?	☐ Never	☐ I leak sometimes when coughing, sneezing, and/or I use a pad when I must exert myself, e.g., sports, work in the garden or yard	☐ I use pads all the time (except possibly during the night), but they are not always wet	☐ I use pads all the time and must change them because they are wet	☐ I leak continuously and need large pads or diapers that must be changed continuously

14. How many pads do you use per 24 hours due to urinary leakage?	☐ I do not use pads<br>☐ Less than 1 per 24 hours<br>☐ Approximately 1 per 24 hours<br>☐ Approximately 2 per 24 hours
15. If you were to live the rest of your life with your urinary tract function just as it is now, how would you experience this?	☐ It would not bother me at all<br>☐ It would bother me a little<br>☐ It would bother me moderately<br>☐ It would bother me very much

Questions about bowel function, ***during the past month:***

	Not at all	A little	Moderately	Much/Very
16. Are you happy with how your bowel works?	☐	☐	☐	☐
17. Do you experience urgency to defecate?	☐	☐	☐	☐
18. Do you have mucus in your stool?	☐	☐	☐	☐
19. Do you have blood in your stool?	☐	☐	☐	☐
20. How much faecal leakage do you experience?	☐	☐	☐	☐

21. Do you have faecal incontinence?	☐ Never	☐ I leak sometimes when I cough, sneeze laugh, lift heavy or when I stand up from a sitting position	☐ I leak when letting gas	☐ I use a pad/diaper continuously that must be changed because they are dirty	☐ I leak continuously and need large pads or diapers that must be changed continuously

22. How many pads do you use per 24 hours due to faecal leakage?	☐ I do not use pads<br>☐ Less than 1 per 24 hours<br>☐ Approximately 1 per 24 hours<br>☐ Approximately 2 per 24 hours
23. If you were to live the rest of your life with your defecation function just as it is now, how would you experience this?	☐ It would not bother me at all<br>☐ It would bother me a little<br>☐ It would bother me moderately<br>☐ It would bother me very much

Questions about your sexual life, ***during the past month***

	Not at all	A little	Moderately	Much/Very
24. Are you happy with your sexual life?	☐	☐	☐	☐

25. Do you have a partner?	☐ Yes	☐ No
26. Are you sexually active (with or without a partner)?	☐ Yes, go to **question 28a**	☐ No

NPCR Nationella prostatacancerregistret — REGIONALA CANCERCENTRUM I SAMVERKAN

27. If you are not sexually active, what is the reason? Answer the question and then proceed to **question 30**	☐ Little or no desire<br>☐ Problems with erection<br>☐ My partner has little or no desire<br>☐ Other cause/causes

28a. Have you used some kind of potency restoration method for sexual activity?	☐ No<br>☐ Yes, self-injection treatment (e.g. Caverject)<br>☐ Yes, substance inserted into the urethra (e.g. Bondil)<br>☐ Yes, pills (e.g. Viagra, Cialis, Levitra)<br>☐ Yes, vacuum pump<br>☐ Yes, other<br><br>28b **If Yes, how often?**<br>☐ Sometimes ☐ Most of the times ☐ Always

29. How is your erection? (Answer even if you use Viagra, Sildenafil or Cialis)	☐ Non-existent<br>☐ Insufficient for any kind of sexual activity<br>☐ Sufficient for masturbation and foreplay<br>☐ Sufficient for intercourse

Mark with ☒ the alternative best describing your situation regardless of whether you use any potency restoration method
Mark only one alternative per question.

	Very weak or non-existent	Weak	Median	Strong	Very strong
30. How would you asses your faith in getting and keeping an erection the past month?	☐ 1	☐ 2	☐ 3	☐ 4	☐ 5

31. How often after sexual stimulation has your erection, during the past month, been enough for penetration?	No sexual activity has occurred	Never or almost never	Less than half of the times	Half of the times	More than half of the times	Almost always or always
	☐ 0	☐ 1	☐ 2	☐ 3	☐ 4	☐ 5

32. How often have you, during intercourse, been able to keep your erection after penetration the past month?	No attempts of intercourse have occurred	Almost never or never	Less than half of the times	Half of the times	More than half of the times	Almost always or always
	☐ 0	☐ 1	☐ 2	☐ 3	☐ 4	☐ 5

33. How difficult have you found it to keep your erection until the end of the intercourse the past month?	No attempts of intercourse have occurred	Very great difficulties	Great difficulties	Difficult	Some difficulties	No difficulties
	☐ 0	☐ 1	☐ 2	☐ 3	☐ 4	☐ 5

SATISFACTION<br>34. When you have tried to have intercourse in <u>the past month</u>, how often have you experienced it as satisfying?	No attempts of intercourse have occurred	Almost never or never	Less than half of the times	Half of the times	More than half of the times	Almost always or always
	☐ 0	☐ 1	☐ 2	☐ 3	☐ 4	☐ 5

35. If you were to live the rest of your life with your sexual function just as it now is, how would you experience this?	☐ It would not bother me at all<br>☐ It would bother me some<br>☐ It would bother me moderately<br>☐ It would bother me much

Thank you for your cooperation!

FOR MORE INFORMATION ABOUT THE QUESTIONNAIRE CONTACT:

Your treating clinic or: National Prostate Cancer Register web page: www.npcr.se
